# Insights into Brain Architectures from the Homological Scaffolds of Functional Connectivity Networks

**DOI:** 10.3389/fnsys.2016.00085

**Published:** 2016-11-08

**Authors:** Louis-David Lord, Paul Expert, Henrique M. Fernandes, Giovanni Petri, Tim J. Van Hartevelt, Francesco Vaccarino, Gustavo Deco, Federico Turkheimer, Morten L. Kringelbach

**Affiliations:** ^1^Hedonia Research Group, Department of Psychiatry, University of OxfordOxford, UK; ^2^Department of Neuroimaging, Institute of Psychiatry, King's College LondonLondon, UK; ^3^Center for Music in the Brain, Aarhus UniversityAarhus, Denmark; ^4^Institute for Scientific Interchange (ISI Foundation)Torino, Italy; ^5^Department of Mathematical Sciences, Politecnico di TorinoTorino, Italy; ^6^Center for Brain and Cognition, Universitat Pompeu FabraBarcelona, Spain; ^7^Instituci Catalana de la Recerca i Estudis Avanats, Universitat Pompeu FabraBarcelona, Spain

**Keywords:** functional connectivity, fMRI, persistent homology, homological scaffold, integration and segregation

## Abstract

In recent years, the application of network analysis to neuroimaging data has provided useful insights about the brain's functional and structural organization in both health and disease. This has proven a significant paradigm shift from the study of individual brain regions in isolation. Graph-based models of the brain consist of vertices, which represent distinct brain areas, and edges which encode the presence (or absence) of a structural or functional relationship between each pair of vertices. By definition, any graph metric will be defined upon this dyadic representation of the brain activity. It is however unclear to what extent these dyadic relationships can capture the brain's complex functional architecture and the encoding of information in distributed networks. Moreover, because network representations of global brain activity are derived from measures that have a continuous response (i.e., interregional BOLD signals), it is methodologically complex to characterize the architecture of functional networks using traditional graph-based approaches. In the present study, we investigate the relationship between standard network metrics computed from dyadic interactions in a functional network, and a metric defined on the *persistence homological scaffold* of the network, which is a summary of the persistent homology structure of resting-state fMRI data. The persistence homological scaffold is a summary network that differs in important ways from the standard network representations of functional neuroimaging data: (i) it is constructed using the information from all edge weights comprised in the original network without applying an *ad hoc* threshold and (ii) as a summary of persistent homology, it considers the contributions of simplicial structures to the network organization rather than dyadic edge-vertices interactions. We investigated the information domain captured by the persistence homological scaffold by computing the strength of each node in the scaffold and comparing it to local graph metrics traditionally employed in neuroimaging studies. We conclude that the persistence scaffold enables the identification of network elements that may support the functional integration of information across distributed brain networks.

## 1. Introduction

The application of graph theoretical analysis to neuroimaging data has provided important new insights about the functional organization of the human brain in health and disease. Graph measures considering the global properties of brain networks have notably helped shape our understanding of the system-wide functional architectures which enable the brain to balance the segregation and integration of information in macro-scale networks (Bullmore and Sporns, 2009, 2012). Complementary to these system-wide characteristics, local graph metrics have been used to quantify the relative importance of individual brain areas toward routing information in brain networks according to different criteria (Section 2.3).

Whilst standard graph metrics are powerful descriptive means to characterize functional neuroimaging data at the whole-brain scale, they also involve significant conceptual and methodological limitations. First, these measures are exclusively based on *dyadic* (i.e., pairwise) interactions between edges and vertices. In practice, this means that the basic “unit” of the graph is an edge connecting a pair of nodes. By contrast, it is well established that neural computations performed by distributed ensembles of brain regions underlie higher cognitive phenomena and even resting-state dynamics in the human brain. As described in detail below, methods from *algebraic topogology* provide an alternative for encoding such non-dyadic relationships. Specifically, the concept of *simplicial complexes* allows one to describe relations between distributed subpopulations of network elements without sacrificing access to many of the fundamental tools of network science (Giusti et al., 2016).

Secondly, the adjacency matrices which form the basis for constructing network representations are derived from measures that have a continuous response and are therefore typically weighted, fully connected, and signed. That is, the value of the pair-wise measure of association (i.e., bivariate/partial correlation, phase synchrony, transfer entropy, mutual information) between the activity signals across brain areas is non-zero, varies considerably across region pairs, and may include both positive and negative values. Therefore, *ad hoc* thresholding methods are commonly employed in functional neuroimaging studies to selectively prune connections within the graph leading to sparser, binary network representations with more naturally interpretable attributes. An exhaustive discussion of the methods used for thresholding brain networks is beyond the scope of this study. It should however be noted that a majority of these strategies lead to the elimination of *weak and/or negative* connections within a network. Yet, it has been demonstrated that standard graph measures are unstable across the threshold ranges typically employed in functional connectivity studies (Garrison et al., 2015) and very few neuroimaging analysis methods actually account for the statistical significance of individual connections (Lord et al., 2011, 2012; Pandit et al., 2013). Thus, while neglecting weak links enhances information clarity, it may well do so at the expense of information completeness. Previous studies have indeed shown weak links to significantly contribute to brain functional processes including: resting-state networks, disease states, and cognition (Schneidman et al., 2006; Schwarz and McGonigle, 2011; Bassett et al., 2012; Cole et al., 2012). Furthermore, synchronous neural oscillations can be maintained even with very weak synaptic links (Buzsáki and Draguhn, 2004) and complex systems research has provided considerable evidence for the contributions of weak links to the stability of large networks in a range of social and biological systems (Granovetter, 1973; Csermely, 2004; Onnela et al., 2007; Pajevic and Plenz, 2012).

**Figure 1:**
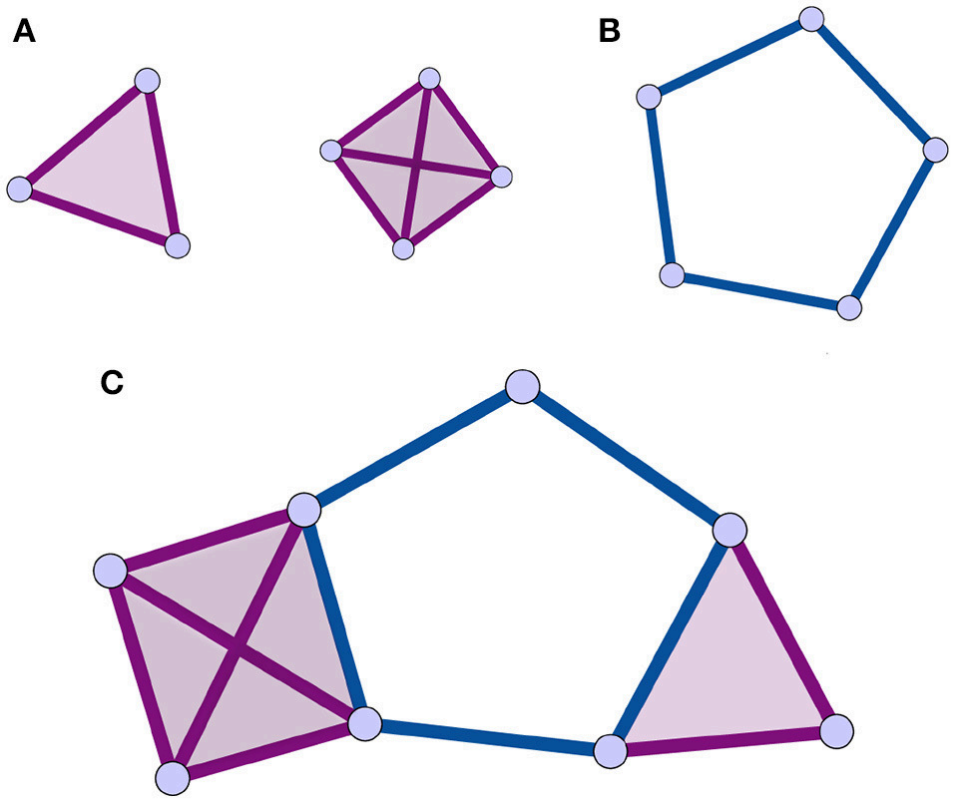
**Illustrations of cliques, simplices, holes, and clique complex**. The simplices are shaded for identification. **(A)** 3 and 4-cliques, which are associated to 2 and 3-dimensional simplices. **(B)** a 1-dimensional hole, or cycle, is a closed path of edges of length greater than 3. **(C)** Combining the elements of **(A,B)** following the rules in Section 2.2.1, one can produce a clique complex with one 1-dimensional hole. All simplices in this figure are shaded as is customary.

An alternative to traditional network analysis methods is the use of the *homological scaffolds* of the weighted network (Petri et al., 2014) to summarize information about the persistent homology of the data. Persistent homology is a recent technique in computational topology (Munkres, 1984; Zomorodian and Carlsson, 2005; Cohen-Steiner et al., 2007) that will be described in detail in Section 2.2. In summary, homology characterizes a topological space by counting its holes of different dimensions (see Section 2.2.2 for definitions). Persistent homology characterizes the importance and stability of the holes in the original data through a process called filtration. It is accordingly a specific type of *mesoscopic organization* of the vertices and edges and their respective importance that is considered in the persistent homology analysis. This enables one to explore the network's organization from a non-dyadic perspective, consistent with the brain's large-scale ensemble coding mechanisms. Holes are the mesoscopic (anti-)structures remaining in the topological space that are not bounding a higher dimensional simplex. The case of 1-dimensional holes, or “cycles,” to which we restrict ourselves in this study, is intuitive to visualize (Figure 1): a cycle is a closed loop of length greater than three.

The network organization of the human brain is characterized by a large number of distributed network modules which perform segregated local computations (Power et al., 2011; Sporns, 2013). There has recently been much interest toward identifying the “hub” regions which enable global communication across segregated brain modules, and the integration of these local computations over space and time (Hansen et al., 2015). The homological scaffolds summarizes the role of network edges constituting the cycles during the filtration process; enabling to identify edges belonging to multiple cycles and/or highly persistent cycles along the filtration. A hypothesis tested in this study is that the edges supporting these mesoscopic network anti-structures will be well positioned to bridge together segregated functional brain modules, rather than participate in densely connected local networks.

The present study investigates the relationship between standard network metrics computed from dyadic interactions in a functional brain network, and a novel metric computed on the *persistence homological scaffold* of the network. Toward this aim we generate a persistence scaffold from the whole-brain functional connectivity data of healthy subjects recorded during resting-state fMRI. We then convert edge-persistence scaffold values into a node-level measure termed *persistence scaffold strength* (*PSS*) which enables comparisons between the persistence scaffold and local graph metrics computed on the original network. We introduce this new measure because homological scaffold theory does not yet include node-level metrics analogous to the topological centrality measures typically used in the analysis of functional brain networks. We find that the unique mathematical attributes of the persistence homological scaffold may render it useful for identifying key local nodes supporting the global integration of information processing directly from functional neuroimaging data.

## 2. Materials and methods

### 2.1. Data

#### 2.1.1. Study participants

Neuroimaging data were collected at CFIN, Aarhus University Hospital, Denmark, from 16 healthy right-handed participants (11 men and 5 women, mean age: 24.7 ± 2.5). Participants with a history of psychiatric or neurological disorders were excluded from participation in the study. The study was previously approved by the Center of Functionally Integrative Neuroscience internal research board. The study was performed in accordance with the Declaration of Helsinki ethical principles for medical research and ethics approval was granted by the Research Ethics Committee of the Central Denmark Region (De Videnskabsetiske Komiter for Region Midtjylland). Informed consent was obtained from all participants.

#### 2.1.2. MRI data acquisition

MRI data were collected in one session on a 3T Siemens Skyra scanner. The parameters for the structural MRI T1 scan were as follows: voxel size of 1 mm^3^; reconstructed matrix size 256 × 256; echo time (TE) of 3.8 ms and repetition time (TR) of 2300 ms. The resting-state fMRI data were collected using whole-brain echo planar images (EPI) with TR = 3030 ms, TE = 27 ms, flip angle = 90^o^, reconstructed matrix size = 96 × 96, voxel size 2 × 2 mm with slice thickness of 2.6 mm and a bandwidth of 1795 Hz/Px. Seven minutes of resting state fMRI data were acquired for each subject.

#### 2.1.3. MRI data processing

We used the automated anatomical labeling (AAL) template (Tzourio-Mazoyer et al., 2002) to parcellate the entire brain into 90 cortical and subcortical regions (45 for each hemisphere) which represented the nodes in functional connectivity networks. The parcellation was conducted in the EPI native space. Linear registration was performed using the FSL toolbox (www.fmrib.ox.ac.uk/fsl, FMRIB, Oxford) (Smith et al., 2004). The EPI image was co-registered to the T1-weighted structural image, and the T1-weighted image was coregistered to the T1 template of ICBM152 in MNI space. The resulting transformations were concatenated and inversed and further applied to warp the AAL template from MNI space to the EPI native space, where interpolation using nearest-neighbor method ensured that the discrete labeling values were preserved. Initial fMRI data preprocessing was carried out using FEAT (FMRI Expert Analysis Tool) Version 6.00, part of FSL and consisted of: motion correction using MCFLIRT; non-brain tissue removal using BET; spatial smoothing using a Gaussian kernel of FWHM 5 mm; grand-mean intensity normalization of the entire 4D dataset by a single multiplicative factor; high pass temporal filtering (Gaussian-weighted least-squares straight line fitting, with sigma = 50.0 s).

#### 2.1.4. Functional connectivity analysis

We used FSL to extract and average the time courses from all voxels within each AAL cluster. We then used Matlab (The MathWorks Inc.) to compute the pairwise Pearson correlation between all 90 regions. *R*-values were transformed to *z*-values via Fisher transformation, and the resulting *z*-values composed the final 90 × 90 functional connectivity (FC) matrix. We averaged the FC matrices for all 16 participants to obtain a group-averaged 90 × 90 FC matrix.

### 2.2. Persistent homology and scaffolds

**Figure 2:**
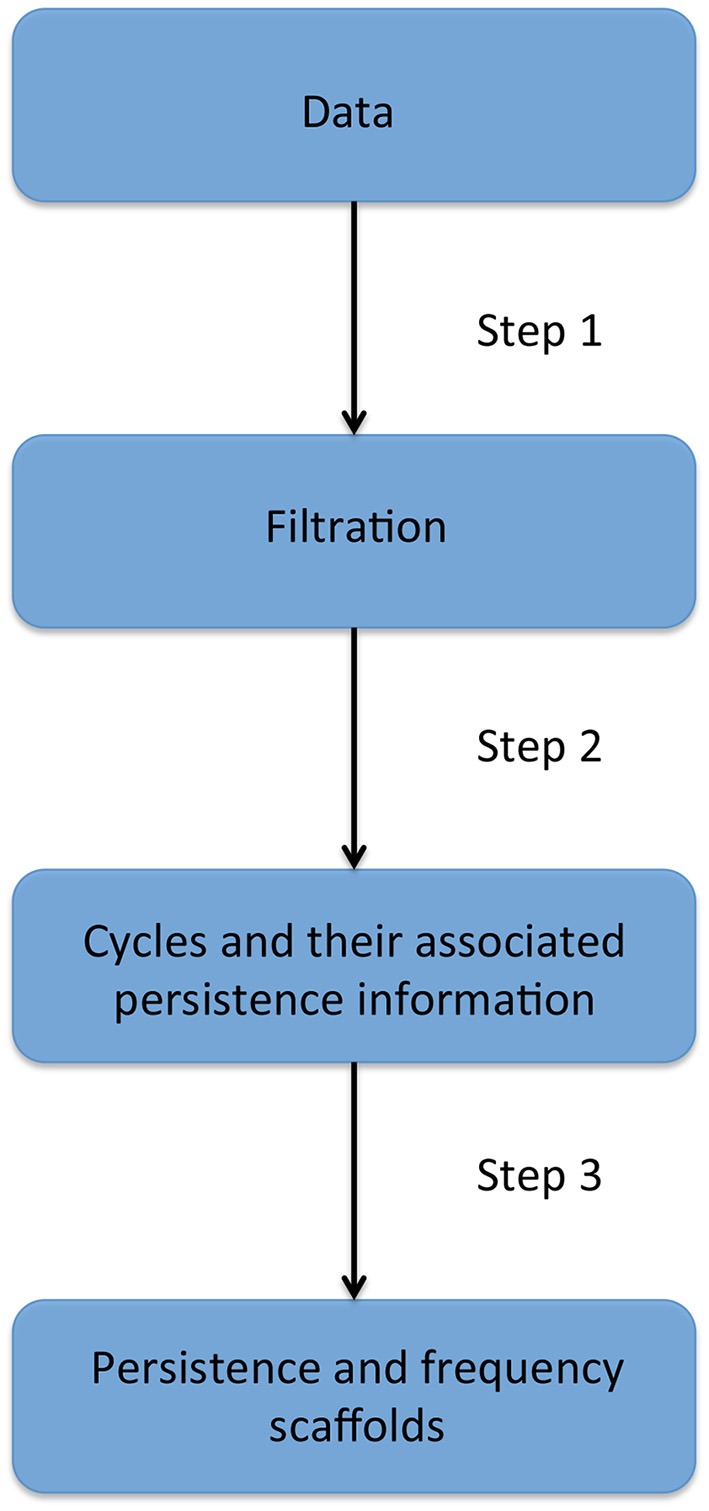
**Description of the four stages of the persistent homology and homological scaffolds analysis workflow**. **The data** consist of a fully connected weighted network. **The filtration** is produced using the weight clique rank filtration. **The persistent homology** of the filtration is computed, and each cycle (or “hole”) is endowed with a birth and death time. **The homological scaffolds** are generated using the information from persistent homology.

The next two sections will introduce fundamental notions needed to understand persistent homology, which is presented in the third section. Homological scaffolds are then defined and a toy example is presented in the penultimate section. The last section exposes the open problem and implications of the choice of a cycle's representative in the filtration. The workflow is illustrated in Figure 2 and can be summarized as follows: one starts from the data, that for the sake of generality we will assume to be a fully connected, weighted, and signed matrix. As the matrix is square and symmetrical, one can interpret it as an undirected network adjacency matrix. The persistent homological features of the data are then computed and finally summarized in the persistence and frequency scaffolds. These scaffolds can be seen as an edge centrality measure, that emphasizes the role of an edge in the persistent homological characterization of the original data but they can also be considered as network in itself and analyzed as such, as we define the *PSS* in Section 2.3.3. For a comprehensive introduction to persistent homology, the interested reader is invited to consult (Munkres, 1984; Zomorodian and Carlsson, 2005; Cohen-Steiner et al., 2007).

#### 2.2.1. Simplices, simplicial complex, and holes

A *simplicial complex* can be seen as a generalization of a graph, where interactions, instead of being strictly between nodes, are between objects called *simplices* that generalize the notion of nodes. In the present context, a node is a 0-dimensional simplex, an edge a 1-dimensional simplex, (representing a binary interaction) a full triangle is a 2-dimensional simplex (representing ternary interactions), and so on for higher dimensions. *A simplicial complex is thus a type of topological space that is a collection of simplices of any dimension* (Figure 1).

There are many types of simplicial complexes. In this study, we focus on *clique complexes*, which can be constructed from any network. In graph theory, a *clique* is a subset of vertices of a graph in which every pair of vertices is adjacent. Thus, a *k*-clique is a completely connected subgraph *K*_*k*_ ⊂ *G*, composed by *k* nodes containing all the possible edges among its nodes. When representing a simplicial complex, simplices are typically shaded, or filled in to identify them (Figure 1). Importantly, upon identifying all the simplices in a clique complex, structures called *holes* can remain, and these are the structures of interest in this analysis (Figure 1). A hole of dimension *k*, or *k*-hole, is a hole bounded by simplices of dimension *k*. In this paper, we focus on holes bounded by 1-dimensional boundaries, also called “cycles.” In a clique complex, a cycle is a minimal closed path of length greater than 3 (Figure 1). This is due to the fact that each clique corresponds to a full simplex so that a triangle is filled in. The set of *k*-holes defining a space is described by the *k*-th homology group *H*_*k*_. Each *k*-hole *i* is in turn represented by its generator $g_{i}^{k}\in H_{k}$. Informally, generators are formed of elements of *H*_*k*_ that identify and can be used to construct the hole.

#### 2.2.2. Homology

One of the most studied problems in mathematics is that of defining a notion of similarity between spaces. Intuitively, two spaces can be thought to be similar if we can transform one into the other via a well-behaved transformation. In particular, if there exists a continuous bijective map, a homeomorphism, that transforms one space into the other, then the two spaces are said to be homeomorphic. Such spaces are, informally, topologically the same, and any of their properties that are conserved by homeomorphism are are thus called *topological invariants*.

The homology group, or simply *homology*, is a property of a space which is based on the counting of holes and their associated dimensions. As an analogy to homology, the reader can think of *The Hound of the Baskervilles* by Sir Arthur Conan Doyle (Doyle, 1998), where the non-manifestation of the hound one night was as informative to Sherlock Holmes as its presence. Homology is a topological invariant which, as explained above, means that it is a property of a space that is preserved by homeomorphisms and keeps the same value whatever the representation of the system (i.e., the bijective map used to look at it). Thus, if two spaces have the same homology, then they are topologically equivalent.

#### 2.2.3. Persistent homology

The process of adding simplices to form a simplicial complex is called a filtration, and the filtration we use in this paper is the *weight clique rank filtration* (Petri et al., 2013). It has been specifically designed to extract homological features from fully connected, weighted and signed networks. The filtration starts with a set of disconnected nodes. Then all the edges from the original network are sorted in descending order of magnitude and added one by one as 1-simplices to the complex. After each addition, the clique complex is constructed and its persistent homology computed. When a new cycle appears, it is tagged with a “birth time,” β_*i*_ and when it disappears, it is tagged with a “death time,” δ_*i*_. The difference between the two time points defines its persistence π_*i*_. It is important to note that when the starting network is fully connected, all the cycles eventually die along the filtration. While it is true that the order in which edges are introduced can depend on very small differences in the weights, the same small differences would alter the persistence or appearance of generators by a similarly small value hence ultimately producing small variations in the scaffold. This is a consequence of the robustness theorems for persistent homology, where one substitutes the usual metric with an extended semi-metric (Cohen-Steiner et al., 2007; Chazal et al., 2012; Bauer and Lesnick, 2014).

#### 2.2.4. Homological scaffolds

The homological scaffolds are secondary networks and were introduced in Petri et al. (2014) as a mean to summarize part of the persistent homology of cycles information for the edges. As they localize the cycles on specific edges of the network, they can naturally be seen as edge centrality measures that characterize the importance of links in the original network through the filtration process, where the weights on the edges represent their centrality.

Two scaffolds are introduced to highlight different aspects of the importance of an edge in the network: the number of cycles an edge belongs to and the total persistence of the cycles it belongs to. The weights of the edges are defined as:

(1) $$\omega_{e}^{f}=\sum_{g_{j}}1_{e\in g_{j}}$$

for the frequency scaffold $H_{G}^{f}$, and

(2) $$\begin{array}{l} \omega_{e}^{p}=\underset{g_{j}|e\in g_{j}}{\sum }\pi_{g_{j}}, \end{array}$$

for the persistence scaffold $H_{G}^{p}$.

The information given by the scaffolds has to be interpreted with care, see Section 2.2.6 below for a full description of the limitations. The python library we developed for persistent homology analysis, that includes the weight rank clique filtration and the scaffolds generation is available at: https://github.com/lordgrilo/Holes.

#### 2.2.5. Example

**Figure 3:**
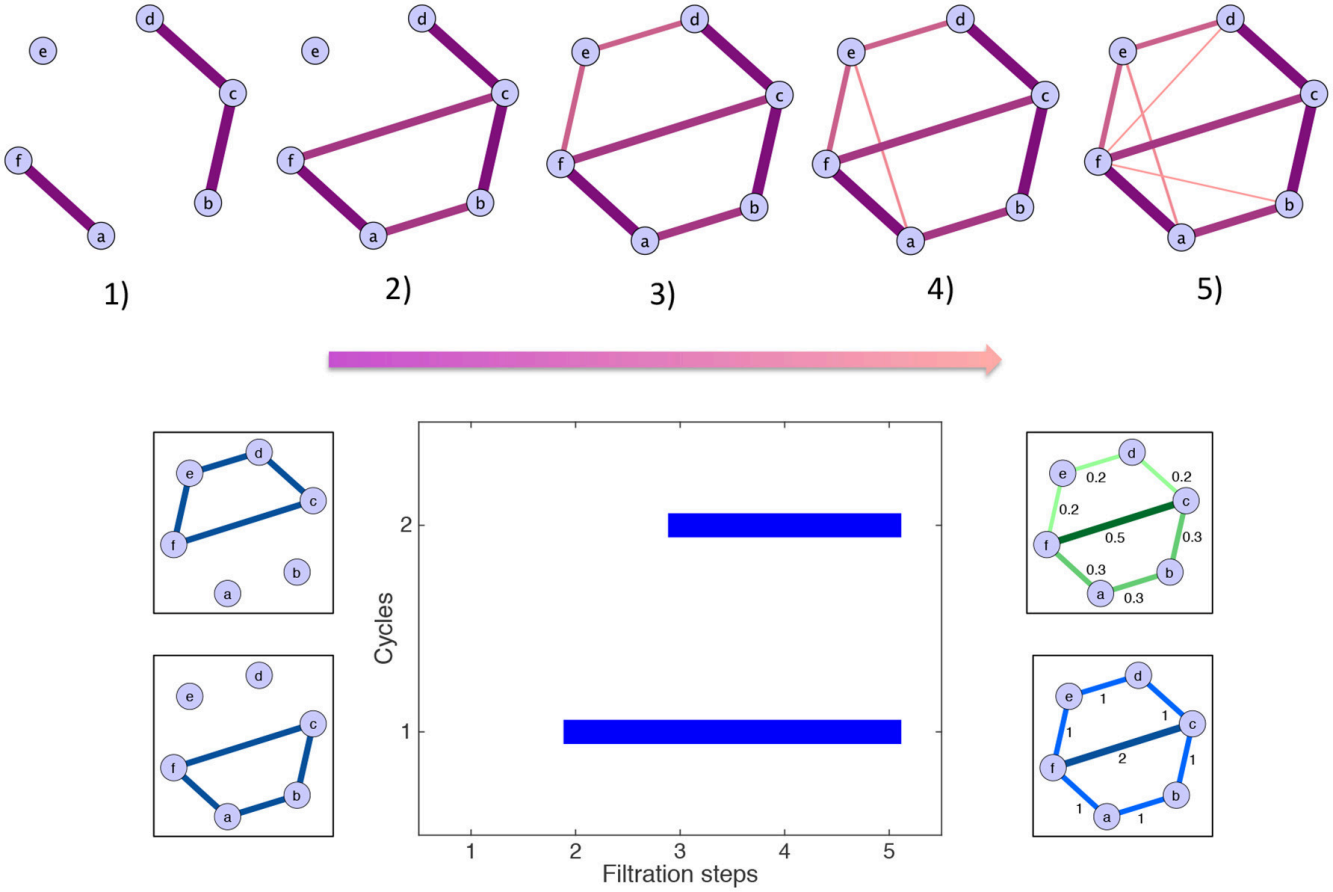
**Toy example illustrating the generation of the homological scaffolds**. **On top** The filtration: edges are added in decreasing order of weight (thickness and color represent the weights) to arrive at the original network at step (5). **Bottom middle** The barcode encoding the persistence of the two cycles 〈*abcf*〉 and 〈*cdef*〉. **Bottom right** The persistence (green) and frequency (blue) scaffolds, summarizing the role of the edges in the cycles present during the filtration.

Persistent homology and the computation of the scaffolds can be illustrated by a simple toy example, which is described in the following lines and shown graphically in Figure 3. For simplicity, some of the edges have a weight of zero and are thus not represented. The first step is the filtration: edges are added in decreasing order of magnitude. In the example, edges have five different weights. Accordingly, five filtration steps are needed, and five associated clique complexes are formed. There are two cycles: one born at step (2) and one born at step (3). By contrast, the edge added at step (4) does not define a new cycle. The aforementioned cycles are both killed by the addition of the two edges at step (5). Their persistences are summarized in the barcode below the filtration. The resulting scaffolds are on the right of the barcode: the persistence scaffold (green) and frequency scaffold (blue). Inspecting the weights of both scaffolds, we conclude that edge 〈*fc*〉 is the most important to support the homological structure of the network.

#### 2.2.6. On the effect of the cycle representative

As illustrated by the present paper and Petri et al. (2014), homological scaffolds can be quite informative, however there is a caveat one has to be aware of when interpreting the results: the choice of a cycle's representative. Persistent homology probes a dataset for its homological features that are persistent—more specifically in the case treated in this paper, cycles. Cycles are topological objects and thus their “sizes” are not uniquely defined, because the homology generators are defined as an equivalence class. Indeed, each cycle corresponding to a certain homology generator can be stretched and deformed, while still remaining a valid representative cycle. In practice, however, to identify homological properties of a topological space, one has to recourse to a representation of the components of the simplices that bound it. In this setting, a hole will be uniquely identified by the edges (or higher-dimensional simplices) forming its smallest boundary at the time of its birth. During the filtration process, a cycle will potentially shrink due to the addition of an edge. Although the shrinking has no topological meaning for the hole itself as it remains the same, its representation changes, i.e., the specific edges forming its boundary change. The question “what is the best representative of a cycle” is an open problem and the definition of *best* strongly depends on the problem at hand.

In practice, however, this will have an impact. We used the software package javaplex (Tausz et al., 2011) in our pipeline for the implementation of persistent homology. It chooses a representative for a cycle and identifies it with the entire lifetime of the cycle. This means that a unique set of edges will represent a cycle, regardless of its possible contraction. This has a direct implication on the scaffolds, and means they are not well-defined. This does not mean they are not informative, but rather that care has to be taken when interpreting the meaning of the particular edges weight forming the scaffolds. The evolution of any cycle representative is a combination of two possible situations:

1. A cycle shrinks by triadic closure,
2. a cycle is split into 2 smaller cycles.

**Figure 4:**
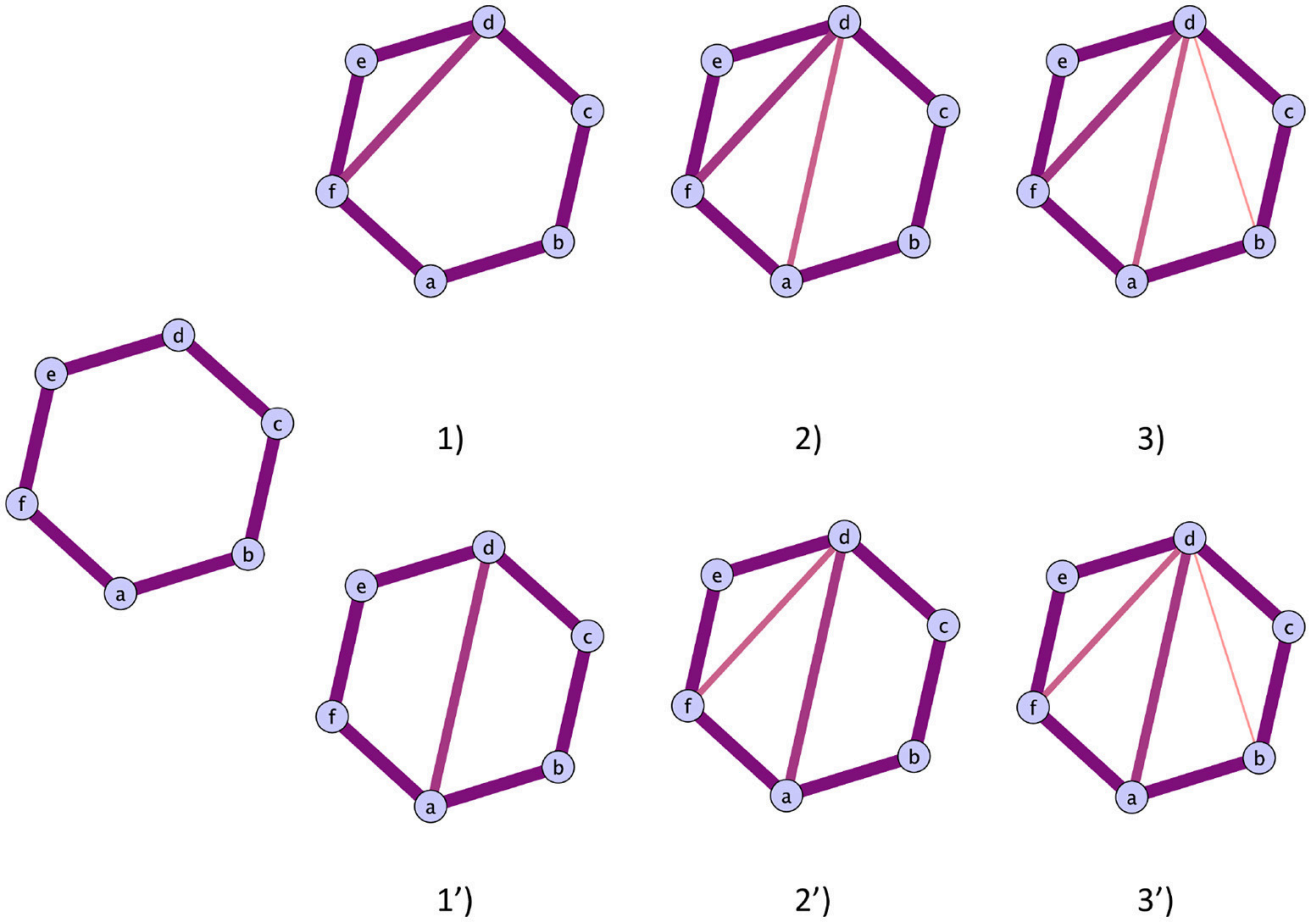
**Illustration of the two possible routes a cycles can close**. **Top route**: The cycles closes with the addition of triangles. The cycles representative will be the original cycles 〈*abcdef*〉, irrespectively of the life time of the sub cycles that are partially closed. **Bottom route**: The original cycle is split into smaller cycles that are eventually closed by the mechanism illustrated in the top route. The two cycles that will be represented in the original cycle 〈*abcdef*〉 and the subcycle 〈*abcd*〉, as the cycle 〈*adef*〉 can be obtained as a linear combination of the first two.

These two possibilities are illustrated in Figure 4, case (i) on the top and case (ii) on the bottom. Therefore, one can monitor the original cycles' subgraphs evolutions as edges are added during the filtration to verify how the cycles die and correctly interpret the homological scaffolds.

Practically, this means exploring the statistics of the holes and verify how they close. It is also important to note that the aforementioned phenomena are more likely to occur in cycles that are long lived.

### 2.3. Graph theoretical analysis

By construction, the graphs that we have considered for the standard graph analysis are unweighted, undirected, and do not contain self-loops. Their adjacency matrix *A* is therefore symmetric, and its elements are equal to 1 if nodes *i* and *j* are connected and zero otherwise.

#### 2.3.1. Standard graph metrics in binarized graphs

We now briefly introduce the standard local centrality measures that were applied to the networks: degree centrality (*DC*), betweenness-centrality (*BC*), local efficiency (*Eff*), and participation coefficient (*PC*). Standard graph measures were calculated using the *Brain Connectivity Toolbox* in Matlab (Rubinov and Sporns, 2010). These metrics each capture different aspects of the contributions of a node to the network organization. To facilitate the interpretation of standard graph metrics, functional connectivity matrices were binarized at eleven statistical thresholds that give a network link density (*D*) in the range [0.10, 0.60] in increments of 0.05, eliminating the weakest links in the network. This thresholding approach was performed using the *threshold_proportional* function of the *Brain Connectivity Toolbox*.

The degree centrality is a measure of the total number of connections that a node has. It therefore depends on the direct neighborhood of the node. For a node *j* within a binarized network comprising *N* nodes, degree centrality is defined as:

(3) $$\begin{array}{l} DC\left(j\right)=\overset{N}{\underset{i=1}{\sum }}A_{i,j} \end{array}$$

The betweenness-centrality of a node measures how many of the shortest paths between all other node pairs pass through it and is a measure of its importance when routing information in the network. By contrast to the degree, *BC* is dependent of the overall topology of the rest of the network beyond the direct neighborhood of a node. For a node *k* it is defined as:

(4) $$\begin{array}{l} BC\left(k\right)=\overset{N}{\underset{i\neq j\neq k,i,j=1}{\sum }}\frac{{\hat{\sigma }}_{i,j}\left(k\right)}{{\hat{\sigma }}_{i,j}} \end{array}$$

where ${\hat{\sigma }}_{i,j}\left(k\right)$ is the number of shortest paths going from node *i* to node *j* through node *k*, and ${\hat{\sigma }}_{i,j}$ is the total number of shortest paths going from node *i* to node *j*.

The local efficiency of a node *k* computes how well the neighbors of a node are connected together. That is, the inverse of the average shortest path length connecting the neighbors of that vertex:

(5) $$Eff(k)=\frac{2}{Nn(n-1)}\sum_{i\in G}^{n}\sum_{i<j\in G}^{n}\frac{1}{d_{i,j}}$$

where *n* is the number of neighbors of a node *k*.

In addition, a community detection algorithm based on modularity (*Louvain method with finetuning*; Blondel et al., 2008) was applied to the adjacency matrix with *D* = 0.40, and identified six communities for the partition optimizing the modularity function. The participation coefficient was then calculated for each node in this network. The participation coefficient compares the degree of a given node to nodes in all other communities with the number of links it has within its own cluster. Nodes with a high participation coefficient are therefore expected to play an important role in binding different communities together and hence contribute to global integration. This measure therefore provides additional information about a node's role in the network topology which cannot be inferred from measures of topological centrality alone. It is defined as:

(6) $$\begin{array}{l} PC_{i}=1-\overset{N_{C}}{\underset{c=1}{\sum }}{\left(\frac{k_{C_{i}}}{k_{i}}\right)}^{2}, \end{array}$$

where *k*_*i*_ is the degree of node *i* and *k_C_i__* its degree limited to cluster *C*.

#### 2.3.2. Weighted network analysis

As a follow-up analysis, we explored the relationship between the *PSS* and the weighted counterparts of the same three graph metrics employed in the original graph analysis described in Section 2.3.1: the nodal *strength* (weighted counterpart of degree), the weighted betweenness centrality (*wt* − *BC*), and the weighted local efficiency (*wt* − *Eff*). By definition, the computation of these measures on a fully connected weighted graph does not rely on the *ad hoc* thresholding of the FC matrix. The mathematical formulation of the weighted version of the metrics are the same as in the unweighted case. For the nodal strength, one sums up the weights of the links connected to a node:

(7) $$\begin{array}{l} SC\left(j\right)=\overset{N}{\underset{i=1}{\sum }}W_{i,j}. \end{array}$$

For the weighted versions of betweenness centrality and efficiency, the difference resides in the definition of the shortest path. In the BCT implementation, the shortest path is computed via a breadth-first search algorithm that follows the links with the smallest weight (Brandes, 2001).

#### 2.3.3. Definition of PSS

Lastly, we define a new centrality measure for the homological scaffolds, the nodal *PSS*. It is essentially the strength of a node, i.e., the sum of the weights of its links, in the persistence scaffold $H_{G}^{p}$. We gave it a different name to clearly differentiate its meaning as a measure obtained from the persistent homology procedure instead of pairwise interactions between edges and vertices. It is defined as:

(8) $$\begin{array}{l} PSS\left(j\right)=\overset{N}{\underset{i=1}{\sum }}H_{G\;i,j}^{p} \end{array}$$

The *PSS* thus compresses into a scalar information about the persistence of cycles passing through a given node. The *PSS* may thereby effectively capture the combination of a nodes central position in the network and the relative lack of connectivity amongst its local neighborhood. Moreover, as outlined above, the *PSS* does not rely on *ad hoc* thresholding of the functional connectivity matrix and therefore includes information from all the edges in the network. This is an important distinction between the *PSS* and the topological centrality metrics traditionally measures applied to functional neuroimaging data.

#### 2.3.4. Definition of functional hubs

Node-level values were calculated for the *PSS* measure as well as standard graph centrality measures. As indicated above, the *PSS* does not require *a priori* thresholding of the functional connectivity matrix. However, for the computation of local graph measures (*DC*, *Eff*, and *BC*), we calculated the node-level metric values at each of eleven different thresholds over the *D* = [0.10, 0.60] range. This curve was then integrated to yield a single nodal metric value that is independent of the threshold. The highest-ranking nodes (termed “hubs” for concision) were then identified for each measure under study. They were defined as those nodes with a metric value larger than 1 standard deviation from the mean of their respective distribution.

## 3. Results

### 3.1. Relationship between nodal *PSS* and standard graph metrics

#### 3.1.1. Topological centrality in binary networks

**Figure 5:**
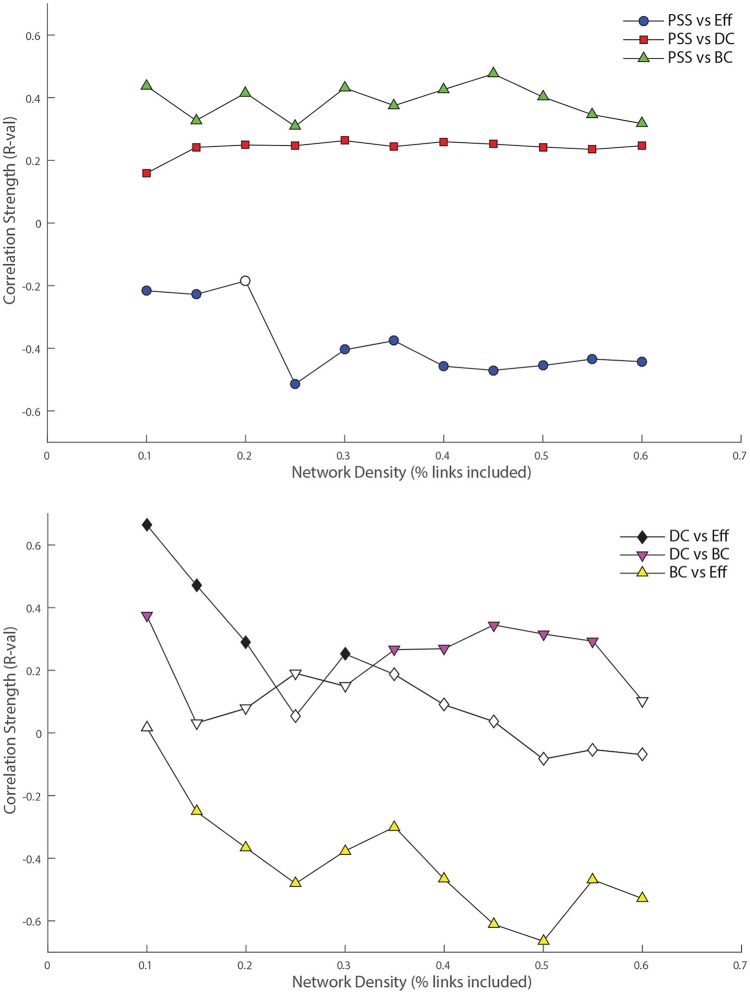
**Top:** Relationship between nodal persistence scaffold strength (*PSS*) and standard topological centrality measures. At each threshold under study, the value of the bivariate correlation coefficient (*R*) between *PSS* and each of: degree-centrality (*DC*), betweenness-centrality (*BC*), and local efficiency (*Eff*) is plotted. **Bottom**: Relationship between standard topological measures. The same procedure as above is repeated for correlations between: *DC* vs. *BC*, *DC* vs. *Eff*, and *BC* vs. *Eff* as control conditions. **Filled shapes** indicate the presence of a **statistically significant** correlation between the two variables (*p* < 0.05).

The main objective of this analysis was to examine the relationship between standard topological centrality measures described above; *DC*, *BC*, *Eff*, and the nodal *PSS*. This was done by computing bivariate correlations between the standard graph metric values and nodal *PSS* across the threshold range applied to the functional connectivity matrix. The *R*-values for each analysis are plotted in Figure 5, and the corresponding *p*-values shown in Table 1. It is important to note that while different FC network thresholds were used for the standard graph analysis, the input FC matrix for the persistent homology analysis did not require *a priori* thresholding, which is a potential strength of this methodology. In order to verify that the reported associations between nodal *PSS* and standard metric values at a given threshold were not simply driven by the direct connectivity of network nodes, we also examined the correlations *DC* vs. *BC*, *DC* vs. *Eff*, and *BC* vs. *Eff* as control conditions (Figure 5).

**Table 1:** *****p***-values of correlations in Figure 5**.

	**PSS v DC**	**PSS v Eff**	**PSS v BC**	**DC v BC**	**DC v Eff**	**BC v Eff**
D = 0.10	0.0001	0.0405	0.0001	0.0003	0.0001	0.8792
D = 0.15	0.0016	0.0306	0.0016	0.7669	0.0001	0.0170
D = 0.20	0.0001	0.0812	0.0001	0.4625	0.0057	0.0004
D = 0.25	0.0031	0.0001	0.0031	0.0725	0.6113	0.0001
D = 0.30	0.0001	0.0001	0.0001	0.1602	0.0165	0.0003
D = 0.35	0.0003	0.0003	0.0003	0.0113	0.0786	0.0041
D = 0.40	0.0001	0.0001	0.0001	0.0104	0.3999	0.0001
D = 0.45	0.0001	0.0001	0.0001	0.0009	0.7328	0.0001
D = 0.50	0.0001	0.0001	0.0001	0.0024	0.4340	0.0001
D = 0.55	0.0009	0.0001	0.0009	0.0051	0.6192	0.0001
D = 0.60	0.0023	0.0001	0.0023	0.3332	0.5165	0.0001

*PSS* vs. *DC*: The positive correlation between *PSS* and *DC* was significant at all thresholds under study, although it was consistently weaker than the correlation of *PSS* vs. *BC*.

*PSS* vs. *BC*: The *PSS* showed strong and also statistically significant positive correlations with the *BC* metric at all thresholds under study. This indicates that *PSS* is associated with a node's tendency to be part of shortest paths between node pairs in the network.

*PSS* vs. *Eff*: Conversely, a strong and significant *negative* correlation was observed between the *PSS* and *Eff* metrics at all but one threshold, showing that high *PSS* nodes generally avoid densely connected neighborhood clusters. These results are illustrated in the top panel of Figure 5.

*DC* vs. *BC*: By contrast to *PSS* vs. *BC*, the *DC* vs. *BC* correlation failed to reach statistical significance at 5 of the 11 thresholds under study. When the relationship did reach statistical significance at some of the higher network densities, the *DC* vs. *BC* correlations remained on average weaker than *PSS* vs. *BC* over the same threshold range.

*DC* vs. *Eff*: The *DC* vs. *Eff* correlation also showed a threshold-dependent profile. Significant positive correlations were observed at some of the lower densities in the *D* = [0.1, 0.2] range which contrasted with the *negative* correlations between *PSS* vs. *Eff* observed at these same thresholds. *DC* vs. *Eff* did not reach statistical significance at any of the thresholds exceeding *D* > 0.35.

*BC* vs. *Eff*: Finally, the negative correlation between the *BC* and *Eff* metrics was qualitatively similar to the *BC* vs. *PSS* correlation over the threshold range. However, *BC* vs. *Eff* did not reach statistical significance at the lowest network density of *D* = 0.1 and the negative correlation strengths at higher densities were overall stronger (and less stable) for *BC* vs. *Eff* than *PSS* vs. *BC*. These results are graphically represented in the bottom panel of Figure 5.

#### 3.1.2. Topological centrality in weighted networks

As a follow-up analysis, the relationships between the *PSS* and the weighted counterparts of the metrics used in the original analysis were also studied. These included the nodal *strength*, weighted betweenness-centrality (*wt* − *BC*) and weighted efficiency (*wt* − *Eff*).

*Strength* vs. *PSS*: There was a borderline significant positive correlation between the nodal strength in the weighted network and the *PSS*: *R* = 0.21, *n* = 90, *p* = 0.046.

*wt* − *BC* vs. *PSS*: The positive correlation between *wt* − *BC* vs. *PSS* was stronger than *strength* vs. *PSS* and highly significant: *R* = 0.39, *n* = 90, *p* < 0.01; consistent with the results of the binary graph analysis.

*wt* − *Eff* vs. *PSS*: There was a significant *positive* correlation between *PSS* vs. *wt* − *Eff*: *R* = 0.23, *n* = 90, *p* = 0.03. This relationship was opposite to that observed in the binary network analysis where *PSS* vs. *Eff* instead showed a strong *negative* association at all thresholds under study.

#### 3.1.3. Participation coefficient

For the network with an intermediate density of *D* = 0.40, a community detection algorithm was applied to the data and the participation coefficient (*PC*) was computed for each node in the network. A significant positive correlation was revealed between *PC* and *PSS*, *R* = 0.32, *n* = 90, *p* < 0.01. This indicated that the *PSS* measure also reflects the tendency of a node to act as a bridge across communities in distributed brain networks.

### 3.2. Identification of functional hubs using the *PSS* and standard graph measures

**Figure 6:**
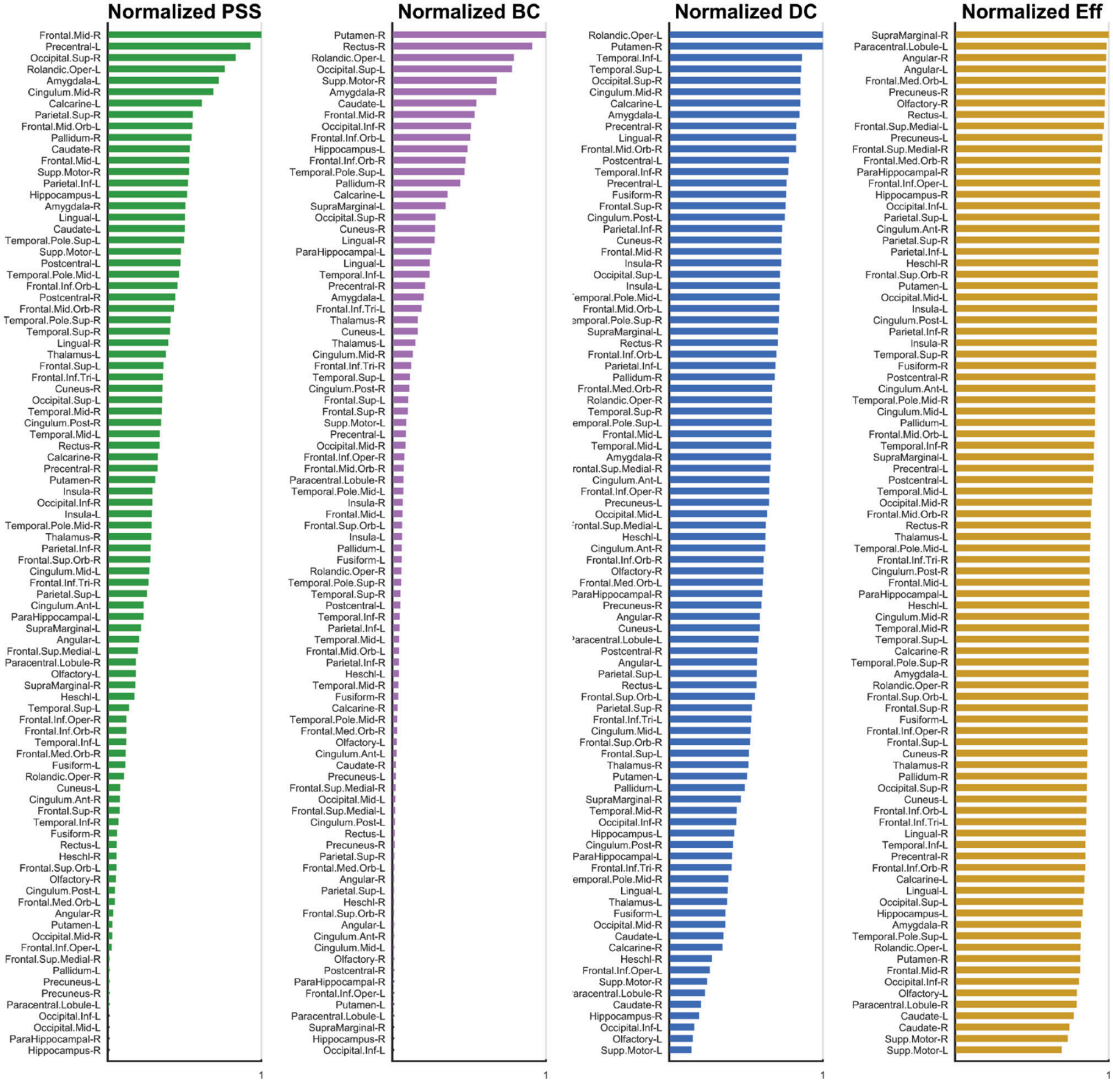
**Normalized Metric Values**. The normalized nodal values are displayed for each graph measure under study. The values for *PSS*, *BC*, *DC*, and *Eff* are respectively depicted from left to right. While computation of the *PSS* does not require *ad hoc* thresholding, the *BC*, *DC*, and *Eff* metrics are threshold-dependent and nodal metric values have thus been integrated over the threshold range under study to generate a single value for each node. The analysis used is described in detail Section 3.2.

**Figure 7:**
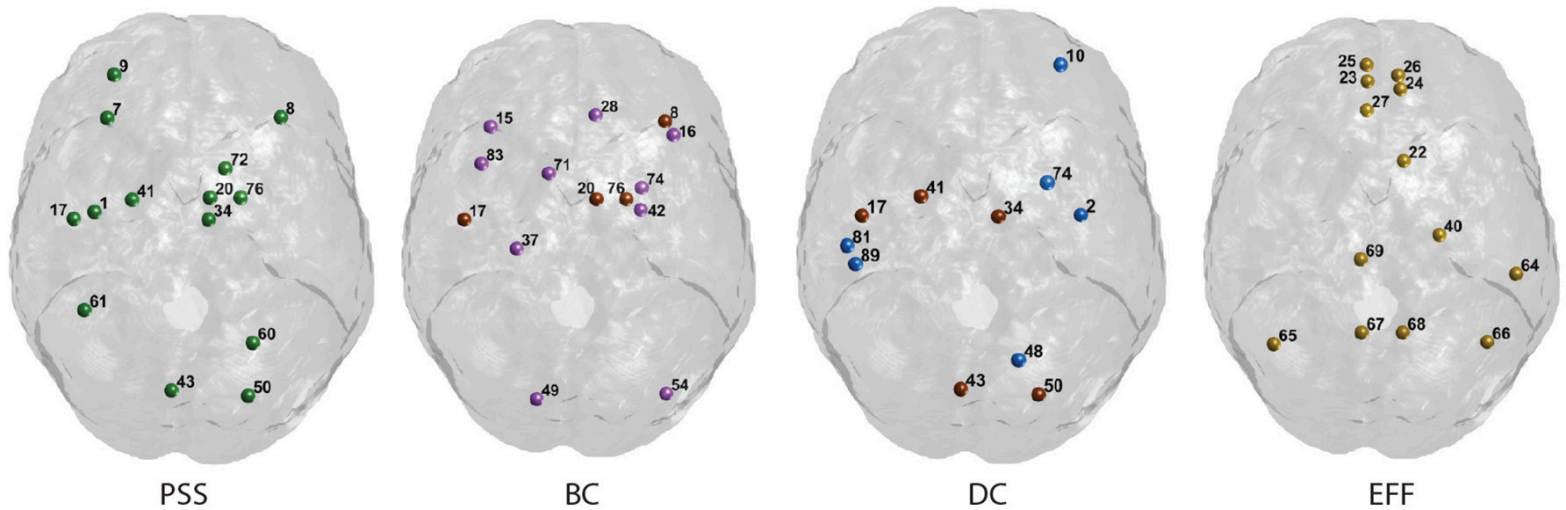
**Graphical display of the highest-ranking nodes**. Functional hubs identified on the *PSS* measure and three standard topological centrality metrics (*BC*, *DC*, *Eff*). Hubs on each measure are defined as having a value >1 S.D. of the mean of their respective distribution. Nodes overlapping with the *PSS* hubs are shown in brown. The corresponding AAL labels for each numerical index are included in Supplementary Figure S1.

We now explain the results shown in Figures 6, 7. Functional hubs were identified on each of the *PSS*, *DC*, *Eff*, and *BC* measures using the procedure outlined in Section 2.3.4. Fourteen AAL regions (out of 90) were identified as hubs on the *PSS* measure. The most important overlap was observed between the *PSS*-hubs and the *DC*-hubs (5/14) and the second-most important overlap was between the *PSS*-hubs and *BC*-hubs (4/14). We note that this was the case despite the presence of a stronger positive correlation between *PSS* vs. *BC* than *PSS* vs. *DC* at all the thresholds under consideration. As expected, *Eff*-hubs showed the least amount of overlap with the *PSS*-hubs, consistent with the strong negative correlation between these two measures.

## 4. Discussion

Persistent homology provides a window into the global organization of the edges' weights fabric of a graph. The present results indicate that persistence homological scaffolds may be useful objects to consider in functional neuroimaging research. The persistence scaffold notably circumvents the need for *ad hoc* thresholding of the functional connectivity matrix and is constructed using the data of all the edges present in the original network. Moreover, the concept of *simplicial complexes* upon which the persistence scaffold is built allows one to describe relations between distributed sub-populations of network elements consistent with the brain's encoding of information in distributed networks, and is not restricted to dyadic associations between region pairs.

In order to study the relationship between standard network metrics and on the persistence homological scaffold, we calculated the strength of each node in the persistence scaffold and termed this novel measure the persistence scaffold strength (*PSS*). The *PSS* measure hence differs in important ways from the standard graph metrics used in neuroimaging studies as it includes information from seemingly unimportant edges with weak weights in the network, and considers the contributions of mesoscopic structures (“cycles”) to the network organization, rather than edge-vertex interactions. We then examined how *PSS* relates to some of the local binarized and weighted graph theoretical metrics typically employed in neuroimaging studies.

Of the binary graph metrics under study, *PSS* showed the strongest positive correlation with the betweenness-centrality metric (*BC*) across the entire threshold range. Even when controlling for the node degree by means of a partial correlation analysis, the positive association between *PSS* and *BC* remained highly significant. This suggested that high *PSS* nodes are likely to contribute to the binding of information across different sources in the brain by creating shortest paths between node pairs. Conversely, a strong negative correlation was observed between *PSS* and local efficiency (*Eff*), and indicates that nodes with a high *PSS* are unlikely to participate in strongly integrated local networks. To further explore the association between the *PSS* measure and functional integration, we conducted a modularity analysis and computed the participation coefficient (*PC*) of network nodes. A strong positive correlation between *PC* and *PSS* was found in the network under study. Nodes with a high participation coefficient preferentially make connections to network communities other than their own, consistent with network roles in global integration.

Taken together, these observations lead to an understanding of the meaning of this new centrality measure and on the interpretation of persistent homological scaffold. The tendency of high *PSS* nodes to bind topologically remote modules in the brain whilst simultaneously avoiding clustered neighborhood reflects the significance of persistent homology in resting-state fMRI data. *PSS* therefore captures different aspects of global network organization in a natural index that does not rely on any weighted average of classic graph metrics, and that extracts this information directly from the data. We also note that although for interpretational purposes we limited ourselves to the study of the first homology group, the *PSS* can easily be generalized to higher dimensions, where it would capture aspects of the network organization that are not reflected at all by traditional network metrics.

When bypassing the thresholding step and instead comparing the *PSS* to the *weighted* counterparts of the standard graph measures computed on the fully connected network, the results for *strength* and *wt* − *BC* were broadly consistent with those of the binarized networks. As in the binary network analysis, the *strength* vs. *PSS* correlation was positive and significant, but weaker than the *wt* − *BC* vs. *PSS* correlation. However, a significant *positive* correlation was observed for the *PSS* vs. *wt* − *Eff* correlation in the weighted network, which was inconsistent with the results of the thresholded network analysis where the binarized version of the two metrics were actually *negatively* correlated at every threshold under study. This exemplifies that the generalization of a binary graph metric to a fully connected weighted network does not imply its specialization.

Finally, we note that the nodal *PSS* does not merely recapitulate the betweenness-centrality metric. Although the correlation between *PSS* and *BC* measures was significant at all thresholds under study in the binary networks analyses, only 4 of the 14 highest ranking *PSS* nodes overlap with the hubs identified on the *BC* metric (Figure 7). This may be explained by the fact that some nodes ranking highly on the betweenness-centrality metric concurrently participate in strongly connected neighborhood clusters; their respective edges would thus form clique complexes at an early stage in the filtration, leading to low *PSS* value. Moreover, the value of the correlation between *PSS* and *BC* was around *R* = 0.4 in both the binarized and weighted network analyses, which further suggests that the *PSS* and *BC* do not reflect identical network attributes.

The highest-ranking regions on the *PSS* measure (Figures 6, 7) were distributed across the brain, consistent with potential roles in the global integration of local networks. There was nevertheless a tendency for the *PSS* hubs to belong to frontal cortical areas (middle and superior frontal gyri, precentral gyrus, rolandic operculum, cingulate), and subcortical structures (amygdala, globus pallidum, caudate nucleus). In the posterior brain, *PSS*-hubs within the parietal lobe included the inferior and superior divisions of the parietal gyrus but did not include midline parietal structures. In the occipital lobe, a visual association area located in the superior occipital cortex ranked highly as a *PSS* hub, as did the calcarine fissure which includes part of the primary visual cortex (V1). We note that V1, which also ranked highly on the *DC* metric in this study, has previously been shown to engage in distributed networks thought to support mental imagery during the resting-state (Wang et al., 2008). Interestingly, no subdivision of the temporal cortices were included amongst *PSS*-hubs, despite several of these regions ranking highly on the *DC* measure.

We also paid attention to the special case of high-ranking *PSS* nodes which did **not** qualify as “hubs” on any of the three standard topological centrality measures (*DC*, *Eff*, *BC*). This subset of nodes was anatomically restricted to the lateral frontal and parietal cortices. They included the middle and superior frontal gyri, as well as inferior and superior sections of parietal gyri. These findings would suggest that, relative to standard topological centrality metrics, the *PSS* may be particularly sensitive to the network activity of frontal and parietal association areas located on the lateral surface of the brain. This would be consistent with the established role of these regions toward supporting high-level cognitive and behavioral functions requiring the large-scale coordination of network elements. The relative importance of *PSS*-hubs toward the information processing capacities of the brain should notably be assessed in future studies by means of virtual lesions in whole-brain computational models (Deco and Kringelbach, 2014; Deco et al., 2015; Váša et al., 2015).

It has now become well recognized that the brain performs local computations in segregated modules that become seamlessly integrated over space and time to support high-level functions necessary for survival. Some brain regions are likely to play a more critical role than others toward enabling the global integration of information. The exact identities of these regions and the optimal experimental approaches for identifying them remain unclear. However, recent evidence would suggest that integrative nodes, such as those potentially identified via the persistence homological scaffold, require metastability for maximal exploration of the full dynamic repertoire of the brain (Kringelbach et al., 2015). Previous research has employed diffusion tensor imaging (DTI) and graph theoretical analysis to identify a subset of hubs which forms a central core or “rich-club” that has been suggested to be important for global brain integration by linking together spatially remote network communities (van den Heuvel and Sporns, 2011). Yet, the mapping of a structural network architecture that can plausibly support segregation and integration does not describe the causal mechanisms and/or activity dynamics that actually underlie functional segregation and integration of information (Deco et al., 2015). The identification of integrator hubs directly from *functional* neuroimaging data using the homological scaffold may be particularly valuable in this regard.

The application of computational topology analysis to functional neuroimaging data is a novel avenue of research, and the physiological significance of homological scaffolds and related measures remains unclear. Given that high *PSS* nodes participate in a large proportion of cycles along the filtration, such nodes may be well positioned to contribute to a specific type of integration where, for example, a given neural pathway diverges than re-converges. Examples of such pathways include the dorsal/ventral visual streams and the well-defined cortico-basal loops between the basal ganglia and motor cortex. Further studies will be needed to test these hypotheses with specificity, but we nevertheless point out that the identification of both visual areas as well as basal ganglia and cortical motor areas amongst the *PSS*-hubs in the present analysis supports this idea.

Whilst the present results suggest that high-ranking *PSS* nodes could be well positioned to support the integration of information across segregated brain modules, further studies will be needed to confirm this observation. One potential approach would be to apply recently developed measures of perturbational integration and segregation in a whole-brain computational model. Previous work has shown that, by perturbing *in silico* neural dynamics by a random set of Gaussian inputs, one can estimate the amount of integration in the system calculated after each perturbation. In this context, perturbational integration is defined by considering the length of the largest connected component of the functional network as an estimate of the amount of integration in the system after each perturbation, as described in detail in Deco et al. (2015). One would therefore expect virtual lesions to high-*PSS* nodes to have a particularly profound impact on the system's integration capabilities, relative to randomly selected network nodes. Another possibility would be to investigate changes in *PSS* hubs assignment and distributions in clinical syndromes characterized by disordered functional integration at the whole-brain scale, such as schizophrenia (Alexander-Bloch et al., 2010; Lynall et al., 2010). Both approaches could help determine to what extent *PSS*-hubs support the integration of network elements, and potentially provide useful insights into the neurobiological attributes of topologically central brain regions in the homological scaffold.

Another limitation of this study, as mentioned in Section 2.2.6, is the choice of the representative cycles for homology classes, which could result in selecting edges that do not belong to the shortest cycle around a certain hole. A possible way around this limitation would be to perform an *a posteriori* analysis of the cycles, in which one controls for the evolution of the subgraph's transitivity (as done in Petri et al., 2014). One could also consider employing computationally cumbersome techniques to track the shortest path across the filtration and then update the scaffold accordingly, Dey et al. (2011a,b). Further work is needed to establish which protocol would be most suited to the specific case of fMRI networks. Our results on network communities nevertheless suggest that the cycle choice issues might not be so critical in our study and potentially lead to a stronger *PSS* interpretation. Indeed network communities, being densely connected internally and strong information integrators, likely constitute the network regions where connected triangle components reside and thus the regions where different representative cycle choices are possible. Moreover, scaffold hubs already tend to have large participation coefficients suggesting that they behave as information brokers between these communities and are therefore, although imperfectly, capturing the large-scale homological structure.

In summary, the present study has explored the relationship between standard network metrics in functional brain network and the persistence homological scaffold derived from the same fMRI dataset. The computation of a local graph measure on the *PSS* differs from standard applications of graph theory to functional neuroimaging data as the scaffolds are not derived from typical dyadic interactions between network elements, and consider information from all edges in the network. The results suggest that topologically central nodes in the persistence scaffold may play important roles toward supporting the functional integration of information across brain modules. Future work should investigate the sensitivity of the homological scaffolds and derived measures to disease-related changes in brain function as well as the specific type of integration performed by the strongest edges and nodes in the scaffolds.

## Author contributions

LDL, PE, MK, FT designed the study. TV, HF, MK collected and processed the fMRI data. PE, GP, FV developed and implemented the persistence homological scaffolds methodology essential to this study. LDL, HF performed the graph theoretical analysis of the data. PE, LDL, TV made the figures. PE, GP, FV, and LDL wrote the methods section. LDL wrote the results section. LDL and PE wrote the introduction and discussion sections, with editorial guidance from MK, FT, and GD.

### Conflict of interest statement

The authors declare that the research was conducted in the absence of any commercial or financial relationships that could be construed as a potential conflict of interest.
