# Supplementary material for: Insights into Brain Architectures from the Homological Scaffolds of Functional Connectivity Networks
Source: Front Syst Neurosci. 2016 Nov 8;10:85. doi: 10.3389/fnsys.2016.00085 (PMC5099524; doi:10.3389/fnsys.2016.00085)
Supplement: Supplementary file 1 [file Image1.pdf]

AAL region	Left	Right
Precentral gyrus	1	2
Superior frontal gyrus, dorsolateral	3	4
Superior frontal gyrus, orbital part	5	6
Middle frontal gyrus	7	8
Middle frontal gyrus, orbital part	9	10
Inferior frontal gyrus, opercular part	11	12
Inferior frontal gyrus, triangular part	13	14
Inferior frontal gyrus, orbital part	15	16
Rolandic operculum	17	18
Supplementary motor area	19	20
Olfactory cortex	21	22
Superior frontal gyrus, medial	23	24
Superior frontal gyrus, medial orbital	25	26
Gyrus rectus	27	28
Insula	29	30
Anterior cingulate and paracingulate gyri	31	32
Middle cingulate and paracingulate gyri	33	34
Posterior cingulate gyrus	35	36
Hippocampus	37	38
Parahippocampal gyrus	39	40
Amygdala	41	42
Calcarine fissure	43	44
Cuneus	45	46
Lingual gyrus	47	48
Superior occipital gyrus	49	50
Middle occipital gyrus	51	52
Inferior occipital gyrus	53	54
Fusiform gyrus	55	56
Postcentral gyrus	57	58
Superior parietal gyrus	59	60
Inferior parietal gyri	61	62
Supramarginal gyrus	63	64
Angular gyrus	65	66
Precuneus	67	68

Paracentral lobule	69	70
Caudate nucleus	71	72
Putamen (lenticular nucleus)	73	74
Pallidum (lenticular nucleus)	75	76
Thalamus	77	78
Heschl's gyrus	79	80
Superior temporal gyrus	81	82
Temporal pole: superior temporal gyrus	83	84
Middle temporal gyrus	85	86
Temporal pole: middle temporal gyrus	87	88
Inferior temporal gyrus	89	90
